# Exploring the Relationship Between Dietary Phytochemical Index and Chemotherapy‐Related Symptoms: Insights From a Cross‐Sectional Study

**DOI:** 10.1002/fsn3.4568

**Published:** 2024-11-07

**Authors:** Barış Yüksel, Gözde Dumlu Bilgin, Hasan Kaan Kavsara

**Affiliations:** ^1^ Department of Nutrition and Dietetics Yeditepe University, Institute of Health Sciences Istanbul Türkiye; ^2^ Faculty of Health Sciences, Department of Nutrition and Dietetics Yeditepe University Istanbul Türkiye; ^3^ Graduate School of Health Sciences, Nutrition and Dietetics Istanbul Medipol University Istanbul Türkiye

**Keywords:** cancer, chemotherapy, dietary phytochemical index, symptoms

## Abstract

Phytochemicals may confer substantial benefits in alleviating chemotherapy‐related symptoms. This cross‐sectional study aimed to determine the role of dietary phytochemicals on treatment‐related symptoms in patients receiving chemotherapy. Data including demographic variables, anthropometric measures such as weight and height, 3‐day food record, and Nightingale Symptom Assessment Scale (N‐SAS), a composite measure of patients' chemotherapy‐related symptoms, were gathered via face‐to‐face interviews. The dietary phytochemical index (DPI) was computed based on the patient's food records and presented by dividing into quartiles. The study included 152 participants with a mean age of 59.59 ± 13.19 years. The mean N‐SAS score was 2.16 ± 0.80. The average DPI score for the entire group was 24.66 ± 6.55, significantly higher in women (26.61 ± 6.06) than men (23.05 ± 6.54) (*p* = 0.001). As the DPI quartile values increased, there was a statistically significant decrease in N‐SAS scores (*p* = 0.002). A significantly negative correlation was found between the N‐SAS score and DPI, as well as all cancers (*r* = −0.364; *p* < 0.001). Additionally, a negative correlation was observed between the N‐SAS score and specific cancer types, comprising lung cancer (*r* = −0.513; *p* = 0.005), breast cancer (*r* = −0.612; *p* < 0.001), and gastrointestinal system (GIS) cancer (*r* = −0.329; *p* = 0.033). Increasing dietary phytochemicals in chemotherapy patients may help manage treatment‐related symptoms. Phytochemicals may confer substantial benefits in alleviating chemotherapy‐related symptoms. This cross‐sectional study aimed to determine the role of dietary phytochemicals on cancer‐related symptoms in patients receiving chemotherapy. A significantly negative correlation was found between N‐SAS score and DPI and all cancers (*r* = −0.364; *p* < 0.001) and different cancer types such as lung (*r* = −0.513; *p* = 0.005), breast (*r* = −0.612; *p* < 0.001), and GIS (*r* = −0.329; *p* = 0.033). As a result, increasing dietary phytochemicals in chemotherapy patients may help manage treatment‐related symptoms.

## Graphical abstract

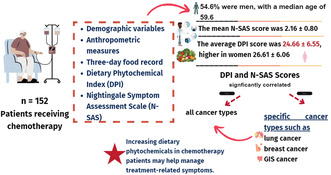

## 1 Introduction

According to the National Cancer Institute (NCI), cancer is characterized by uncontrolled growth and dissemination of abnormal cells throughout the body (Brown et al. 2023; National Cancer Institute n.d.). Despite significant advancements in chemotherapy and other treatment modalities, cancer continues to be a burden, causing substantial physical distress for patients and considerable economic strain on families and healthcare systems worldwide (Carrera, Kantarjian, and Blinder 2018).

Globally, the incidence of cancer remains alarmingly high, with an estimated 19,965,054 cases reported across both genders, with lung cancer ranking as the most prevalent type, accounting for 2,480,308 cases (12.4%) (Globocan WHO 2022). In Türkiye, the year 2022 documented 240,013 reported cases, with lung cancer topping the list at 41,032 cases (17.1%), followed by breast cancer (25,249, 10.5%), colorectal cancer (21,718, 9.0%), prostate cancer (17,274, 7.2%), thyroid cancer (15,376, 6.4%), and bladder cancer (13,125, 5.5%) (Globocan WHO 2022).

While chemotherapy remains a cornerstone of cancer management, its efficacy is often impeded by severe adverse effects, ranging from myelosuppression to neurotoxicity, frequently necessitating treatment discontinuation. Remarkably, a study conducted in Türkiye highlighted that the most commonly reported side effects of chemotherapy included nausea and vomiting (79.3%) and fatigue (74.7%), with other notable symptoms including decreased appetite (65.5%), changes in taste (60.9%), hair loss (60.0%), dry mouth (51.7%), and constipation (51.7%) (Altun and Sonkaya 2018). Due to the high prevalence of these adverse effects, it is imperative to investigate complementary approaches that can alleviate chemotherapy‐induced symptoms and improve treatment outcomes (Lustberg et al. 2023).

In recent years, there has been an increasing interest in the potential therapeutic benefits of natural compounds, specifically phytochemicals derived from various dietary sources, as adjunctive therapies in cancer treatment. These bioactive compounds offer promising ways to alleviate chemotherapy‐related symptoms and improve therapeutic effectiveness due to their multifaceted pharmacological properties, such as antioxidant (Turan et al. 2023), anti‐inflammatory (Saleh et al. 2023), antimicrobial (Dalal et al. 2023), and anticancer effects (Liu et al. 2021). A noteworthy study has underscored the significance of DPI in modulating cancer risk, with findings suggesting a negative association between DPI and breast cancer risk (Acikgoz Pinar, Yildiz, and Altundag 2023).

Furthermore, Sundaram et al. (2019) demonstrated that combining phytochemicals and chemotherapeutic agents have enhanced therapeutic potential against various cancer types, particularly in human cervical cancer cells. Notably, dietary patterns among cancer patients, particularly pediatric populations, have revealed suboptimal intake of polyphenols during cancer treatment, highlighting the potential therapeutic role of polyphenols in pediatric oncology (Liu, Cohen, and Vittorio 2019). Similarly, synergistic interactions among phytochemicals have inhibited prostate cancer cell proliferation (Brahmbhatt et al. 2013), emphasizing the importance of a holistic approach to dietary interventions in cancer management.

Our study aims to evaluate the relationship between DPI ‐that is a measure of the intake of phytochemicals, which are bioactive compounds found in plant‐based foods known for their potential health benefits, including anti‐inflammatory and antioxidant properties‐ and severity of chemotherapy‐related symptoms experienced by cancer patients across diverse cancer types. Chemotherapy‐related symptoms assessed in this study include physical well‐being (e.g., fatigue, nausea, mouth sensitivity, changes in taste), social well‐being (e.g., alopecia, skin changes, forgetfulness), and psychological well‐being (e.g., difficulties in daily activities, social withdrawal, anger). This study recognizes the significance of nutrition in cancer care and seeks to elucidate potential dietary interventions to enhance patients' quality of life and treatment outcomes. To our knowledge, this study represents the first attempt to assess the correlation between DPI and symptom severity in cancer patients undergoing chemotherapy.

## 2 Materials and Methods

### 2.1 Study Design

This single‐center cross‐sectional study was performed on cancer patients admitted to the Oncology Clinic of a private hospital in İstanbul, Türkiye between January 2023 and June 2023. Inclusion criteria for the study required participants to be 18 years of age or older, diagnosed with cancer, and receiving chemotherapy as a treatment. Pregnant or breastfeeding women, participants on a special diet (diabetic, gluten‐free, etc.) in the past year, and participants with severe chronic diseases such as renal or hepatic failure were excluded from the present study.

This study was conducted in accordance with the guidelines set forth in the Declaration of Helsinki, and all procedures were approved by the Noninvasive Research Ethics Committee of Yeditepe University on date 11.03.2022 with the number 202203Y0198. Prior to participation in the study, all participants read and signed a written informed consent form.

### 2.2 Data Collection Form

The data were gathered based on the self‐reports of participants who had volunteered to take part in the study, had received chemotherapy, and had visited the diet clinic on a regular basis. The data collection form consists of three parts: (i) sociodemographic characteristics (gender, marital status, childbearing status, education level, employment status, income level, smoking, and alcohol consumption status), (ii) cancer‐related information (types of cancer, cancer stage, weight fluctuations after diagnosis), and (iii) anthropometric measurements.

### 2.3 Anthropometric Measurements

Anthropometric measurements, including weight and height, were collected by a trained dietitian in accordance with World Health Organization (WHO) guidelines (World Health Organization 2022) and were repeated twice. In an effort to minimize the margin of error, the patients' measurements were taken before chemotherapy. The participants' body weights were measured with a precision of 0.1 kg using a Tanita SC‐330 digital medical scale, and their height was measured without shoes in a standing position using a stadiometer with an accuracy of 0.1 cm. Then, the body mass index (BMI) was computed using the metric formula: weight (kg)/height (m^2^) and categorized according to World Health Organization criteria as underweight (< 18.5 kg/m^2^), normal (18.5–24.99 kg/m^2^), overweight (25.0–29.99 kg/m^2^), or obese (≥ 30.0 kg/m^2^) (World Health Organization 2022).

### 2.4 Three‐Day Food Record

A trained dietitian obtained the participants' dietary intake through the 3‐day food record including 2 weekdays and 1 weekend day. Patients were instructed to note the quantities of food consumed using standard measuring instruments, such as water glasses and tablespoons, in order to facilitate the accurate documentation of food intake and ensure uniformity in the recording of these measurements. All food‐related data were analyzed using the Computer Assisted Nutrition Program, Nutrition Information System (BeBis 8.2), developed for Türkiye. The participants' total energy and energy intake from phytochemical‐rich foods were analyzed to calculate DPI.

### 2.5 Nightingale Symptom Assessment Scale (N‐SAS)

The scale developed by Can and Aydiner (2011) determines the well‐being levels of individuals diagnosed with cancer in accordance with the symptoms they experience during the chemotherapy process. It consists of a total of 38 items with three subdimensions: Physical Well‐being (PhWB) (20 items), Social Well‐being (SoWB) (8 items), and Psychological Well‐being (PsWB) (10 items).

Items are scored on a five‐point Likert scale ranging from 0 to 4, with “very good well‐being” between 0 and 0.50, “good well‐being” between 0.51 and 1.50, “moderate well‐being” between 1.51 and 2.50, “poor well‐being” between 2.51 and 3.50, and “very poor well‐being” between 3.51 and 4.00. Total and subscale scores are obtained by adding the scores given to the items and dividing them by the number of items. High scores indicate a negative overall quality of life and subdimensional well‐being. In the development study, the Cronbach alpha reliability coefficient was 0.93 for the total scale and varied between 0.81 and 0.87 for the subscales.

### 2.6 Dietary Phytochemical Index (DPI)

The DPI emerged in 2004 with the proposal of Mark F. McCarty (McCarty 2004) to determine the phytochemical content of the diet. According to this approach, DPI encompasses phytochemical‐rich food components such as fruits, vegetables, natural fruit and vegetable juices, whole grains, legumes, nuts, seeds, olives, and olive oil, alcoholic beverages such as wine, beer, and cider (except distilled spirits), and soy products (soybeans). Potatoes, pickled, and powdered vegetables were not included in the analysis as they are not considered a rich source of phytochemicals.

The DPI was calculated using the following formula based on the patients' three‐day food records.

$$\begin{array}{c} \mathrm{DPI}=\left[\text{Total energy from phytochemical}-\text{rich foods}\;\left(\text{kcal}/d\right)/\right.\\ \;\left.\text{total energy intake}\;\left(\text{kcal}/d\right)\right]\times 100. \end{array}$$

### 2.7 Statistical Analysis

Since approximately 50 cancer patients receiving chemotherapy were registered each month, 250 patients were considered as the population. The targeted minimum sample size was computed to be 151 (*d* = 0.05, *p* = 0.05, *q* = 0.05), and at the end of the 5‐month data collection process, 152 patients were included in the study. All statistical analyses were performed with the IBM SPSS Statistics software (version 23), and the results were considered statistically significant for *p* < 0.05. The quantitative variables of participants were presented as mean and standard deviation, while qualitative variables were presented as frequency (*n*) and percentage (%). To perform advanced analysis, we categorized DPI values into quartiles: Q1 (< 19.73), Q2 (19.73–23.70), Q3 (23.71–29.91), and Q4 (> 29.91). Q4 is more favorable and highest score of DPI. For comparison of these values between the categorical and continuous characteristics of the participants, the Pearson χ^2^ test or independent *t*‐test were used, respectively. The Pearson correlation coefficients (*r*) were calculated to determine the relationship between the DPI and N‐SAS among different types of cancer.

## 3 Results

This study was designed to highlight the role of the phytochemical content of the diet on treatment‐related symptoms of patients who received chemotherapy treatment. The total sample included 152 participants with cancer, and the descriptive characteristics of respondents are depicted in Table 1. Most participants were men (54.6%, *n* = 83), with a mean age of 59.59 ± 13.19 years. 86.8% (*n* = 132) were married and 36.8% (*n* = 56) had bachelor's degree. Most of those involved in the study were retired, had more income than expenses, and did not smoke or drink alcohol (Table 1). The BMI of the participants ranged from 15.6 to 41.9 kg/m^2^, with a mean of 24.4 ± 4.3 kg/m^2^. The mean BMI of women (26.48 ± 4.98) was significantly higher than that of men patients (22.83 ± 2.82). Furthermore, overweight and obesity were significantly more common in women (40.6% vs. 21.7%) than in men (18.1% vs. 1.2%) (*p* < 0.001) (Table 2).

**TABLE 1:** General characteristics of participants.

Variables	*n* (%)
Gender	
Men	83 (54.6)
Women	69 (45.4)
Marital status	
Single	20 (13.2)
Married	132 (86.8)
Education	
Illiterate	7 (4.6)
Primary school graduate	17 (11.2)
Secondary school graduate	27 (17.8)
High school graduate	37 (24.3)
Bachelor's degree	56 (36.8)
Postgraduate degree	8 (5.3)
Employment status	
Housewife	41 (27.0)
Civil servant	11 (7.2)
Private sector employee	29 (19.1)
Retired	55 (36.2)
Self‐employed	16 (10.5)
Income Status	
Expense > Income	16 (10.5)
Income = Expense	68 (44.7)
Income > Expense	54 (35.5)
Income > more than twice the expenses	14 (9.3)
Smoking status	
Does not smoke	76 (50.0)
Smoke	19 (12.5)
Ex‐smokers	57 (37.5)
Alcohol consumption	
Does not drink	99 (65.1)
Drinks	12 (7.9)
Quit drinking	41 (27.0)

**TABLE 2:** Participants' age and anthropometric measurements by gender.

Variables	Total (*n* = 152)	Men (*n* = 83)	Women (*n* = 69)	*p*
Age (years)	59.59 ± 13.19	61.69 ± 13.63	57.06 ± 12.26	0.031*
Weight (kg)	67.89 ± 10.71	68.77 ± 10.20	66.82 ± 11.28	0.265
BMI (kg/m^2^)	24.49 ± 4.34	22.83 ± 2.82	26.48 ± 4.98	< 0.001**
BMI classification				
Underweight	6 (4.0)	4 (4.8)	2 (2.9)	< 0.001**
Normal	87 (57.2)	63 (75.9)	24 (34.8)	
Overweight	43 (28.3)	15 (18.1)	28 (40.6)	
Obese	16 (10.5)	1 (1.2)	15 (21.7)	

*Note:* BMI, Body mass index (Underweight < 18.5 kg/m^2^; Normal: 18.5–24.99 kg/m^2^; Overweight: BMI 25.0–29.99 kg/m^2^; Obesity: BMI ≥ 30 kg/m^2^). Variables are shown as means ± standard deviations (SD). Categorical variables are expressed as frequency (%). *p‐*values were computed by independent *t*‐test for continuous variables and χ^2^ for categorical variables. *p < 0.05, **p < 0.01.

When the study population was examined, it was reported that the most prevalent cancers were GIS, breast, and lung. Concerning gender allocation, while GIS (32.5%), lung (25.3%), and urological (21.7%) cancers are seen in men, this trend is noted in breast (50.7%), GIS (21.7%), and gynecological (13%) cancers in women (Table 3). Apart from gender‐specific cancers, lung cancer (*p* = 0.029) and pancreatic‐hepatobiliary cancer was (*p* = 0.012) found to be significantly more common in men than in women. In addition, the preponderance of participants in the study had stage IV (46.4%) cancer and had been previously operated on (75%). Following diagnosis, there was a predominant tendency for weight loss across all patients. However, this loss was more pronounced in men than women, while the opposite was observed with regard to weight gain, which was more prevalent in women (*p* = 0.002). The average DPI score for the entire group was 24.66 ± 6.55 and was recorded to be significantly higher in women (26.61 ± 6.06) than in men (23.05 ± 6.54) (*p* = 0.001) The mean N‐SAS, a composite measure of patients' chemotherapy‐related symptoms, was 2.16 ± 0.80, but no significant difference was recorded between the women (2.12 ± 0.84) and men (2.21 ± 0.77). Moreover, according to this scale category, almost half of the patients had poor well‐being (42.8%, *n* = 65) (Table 3).

**TABLE 3:** Cancer‐related characteristics of participants by gender.

Variables	Total (*n* = 152)	Men (*n* = 83)	Women (*n* = 69)	*p*
Types of cancer *n* (%)				
Lung	28 (18.4)	21 (25.3)	7 (10.1)	0.029*
Breast	35 (23.0)	0 (0)	35 (50.7)	< 0.001**
Gynecological	9 (5.9)	0	9 (13)	0.001**
GIS	42 (27.6)	27 (32.5)	15 (21.7)	0.194
Head–Neck	8 (5.3)	7 (8.4)	1 (1.4)	0.072
Urological	19 (12.5)	18 (21.7)	1 (1.4)	< 0.001**
Pancreatic‐hepatobiliary	11 (7.2)	10 (12.0)	1 (1.4)	0.012*
Cancer Stage				
I	11 (6.6)	4 (4.8)	7 (10.2)	0.155
II	53 (35.1)	26 (31.3)	27 (39.1)	
III	18 (11.9)	14 (16.9)	4 (5.8)	
IV	70 (46.4)	39 (47.0)	31 (44.9)	
Radiotherapy				
Yes	54 (35.5)	31 (37.3)	23 (33.3)	0.730
No	98 (64.5)	52 (62.7)	46 (66.7)	
Surgery				
Yes	114 (75.0)	59 (71.1)	55 (79.7)	0.301
No	38 (25.0)	24 (28.9)	14 (20.3)	
Weight fluctuations after diagnosis				
Gained weight	20 (13.2)	4 (4.8)	16 (23.2)	0.002**
Lost weight	132 (86.8)	79 (95.2)	53 (76.8)	
N‐SAS Score	2.16 ± 0.80	2.21 ± 0.77	2.12 ± 0.84	0.526
N‐SAS Score Categories				
Good well‐being	38 (25)	19 (22.9)	19 (27.5)	0.729
Moderate well‐being	48 (31.6)	26 (31.3)	22 (31.9)	
Poor well‐being	65 (42.8)	37 (44.6)	28 (40.6)	
Very poor well‐being	1 (0.6)	1 (1.2)	0 (0)	
DPI	24.66 ± 6.55	23.05 ± 6.54	26.61 ± 6.06	0.001**

*Note:* Variables are shown as means ± standard deviations (SD). Categorical variables are expressed as frequency (%). *p*‐values were computed by independent *t*‐test for continuous variables and χ^2^ for categorical variables. *p < 0.05 significant, **p < 0.01.

Abbreviations: BMI, body mass index; DPI, dietary phytochemical index; GIS, gastrointestinal system; N‐SAS, nightingale symptom assessment scale.

Analysis by different types of cancer indicated that the patients with the lowest DPI scores were those with head and neck cancer (17.65 ± 7.20), while patients with the highest DPI scores were those with gynecological cancers (29.81 ± 4.07). When examined according to cancer type, the highest average N‐SAS score, reflecting a negative prognosis, was detected in patients with head and neck cancer (2.76 ± 0.60) (Table 4).

**TABLE 4:** Characteristics of participants according to types of cancer.

Types of cancer	*n*	Weight (kg)	BMI (kg/m^2^)	Weight change	N‐SAS score	DPI
Lung	28	65.89 ± 8.77	22.77 ± 3.30	−11.25 ± 6.61	2.56 ± 0.48	26.54 ± 6.42
Breast	35	68.20 ± 11.05	27.53 ± 4.81	−4.20 ± 5.92	2.02 ± 0.88	28.29 ± 5.33
Gynecological	9	74.44 ± 11.05	29.16 ± 3.87	−0.78 ± 6.55	1.48 ± 0.55	29.81 ± 4.07
GIS	42	67.09 ± 10.89	23.57 ± 3.77	−12.71 ± 7.97	2.24 ± 0.77	21.31 ± 5.10
Head–Neck	8	59.50 ± 5.95	20.86 ± 1.97	−18.25 ± 4.26	2.76 ± 0.60	17.65 ± 7.20
Urological	19	73.97 ± 12.10	24.20 ± 3.17	−8.32 ± 9.54	1.72 ± 0.76	23.86 ± 4.74
Pancreatic‐hepatobiliary	11	65.27 ± 7.11	22.01 ± 1.90	−13.00 ± 4.21	2.23 ± 0.91	23.40 ± 8.58

Abbreviations: BMI, body mass index; DPI, dietary phytochemical index; N‐SAS, nightingale symptom assessment scale.

Further interpretation of the DPI by quartiles is illustrated in Table 5, and while DPI scores tended to increase across quartiles for women, this trend was the opposite for men (*p* = 0.001). Moreover, it was noticed that as the quartile levels increased, there was a statistically significant decrease in N‐SAS scores (2.42 ± 0.88 vs. 1.74 ± 0.69) (*p* = 0.002) and weight change (−13.8 ± 9.2 vs. ‐5.4 ± 8.2) (*p* < 0.001) and an increase in BMI (22.9 ± 3.6 vs. 25.7 ± 4.2) (*p* = 0.021). Table 6 presents the correlations between different types of cancers and N‐SAS score and DPIs. Accordingly, a significantly negative correlation was found between N‐SAS score and DPI and all cancers (*r* = −0.364; *p* < 0.001) and different cancer types such as lung (*r* = −0.513; *p* = 0.005), breast (*r* = −0.612; *p* < 0.001), and GIS (*r* = −0.329; *p* = 0.033).

**TABLE 5:** Characteristics of participants across the quartiles of DPI.

Characteristics	Q1 (*n* = 38)	Q2 (*n* = 39)	Q3 (*n* = 39)	Q4 (*n* = 36)	*p*
DPI score	16.3 ± 2.8	22.1 ± 1.3	27.7 ± 1.6	33.0 ± 2.6	
Age (year)	59.6 ± 13.2	60.5 ± 12.8	62.2 ± 14.2	55.8 ± 12.2	0.201
Gender *n* (%)					
Men	28 (73.7)	24 (61.5)	21 (53.8)	10 (27.8)	0.001**
Women	10 (26.3)	15 (38.5)	18 (46.2)	26 (72.2)	
Weight (kg)	67.0 ± 12.7	70.4 ± 11.9	66.2 ± 8.1	67.9 ± 9.4	0.332
BMI (kg/m^2^)	22.9 ± 3.6	25.3 ± 5.0	24.0 ± 3.9	25.7 ± 4.2	0.021*
Weight change	−13.8 ± 9.2	−10.4 ± 5.8	−8.3 ± 7.3	−5.4 ± 8.2	< 0.001**
N‐SAS Score	2.42 ± 0.88	2.26 ± 0.71	2.23 ± 0.77	1.74 ± 0.69	0.002**

*Note:* Q1 (< 19.73), Q2 (19.73–23.70), Q3 (23.71–29.91), and Q4 (> 29.91). *p*‐values for differences between quartiles of DPI were calculated with one‐way analysis of variance (ANOVA) with post hoc Bonferroni test for comparison between the individual group and variables are presented as means ± standard deviations (SD). Categorical variables are expressed as frequency (%) and were computed by χ^2^. *p < 0.05; **p < 0.01.

Abbreviations: BMI, body mass index; DPI, dietary phytochemical index; N‐SAS, nightingale symptom assessment scale.

**TABLE 6:** Relationship of different types of cancers with N‐SAS score and DPI.

Variables	*n*	*r*_*p*_	*p*
All cancers	152	−0.364	< 0.001**
Lung	28	−0.513	0.005**
Breast	35	−0.612	< 0.001**
Gynecological	9	−0.294	0.443
GIS	42	−0.329	0.033*
Head–Neck	8	−0.547	0.161
Urological	19	0.250	0.301
Pancreatic‐hepatobiliary	11	−0.267	0.427

*Note:* Correlation is significant at *p < 0.05; **p < 0.01.

Abbreviations: GIS, gastrointestinal system; N‐SAS, nightingale symptom assessment scale; *r*_*p*_, Pearson's rank correlation coefficient.

## 4 Discussion

The present study addressed the relationship between DPI and post‐treatment symptoms in cancer patients undergoing chemotherapy. Although cancer patients have many different concurrent symptoms due to chemotherapy, we hypothesized that as the phytochemical content of the diet increases, symptom scores will decrease, and the phytochemical component of the diet may serve as a modulating agent. In accordance with our hypothesis, this study revealed a significant negative correlation between N‐SAS score and DPI in several types of cancer, including lung, breast, and GIS cancers.

Phytochemicals affect various aspects of chemotherapeutic regimens, and their incorporation into the treatment of cancer patients is warranted and necessary. These natural compounds provide numerous potential benefits, such as improving the efficacy of chemotherapeutic substances, decreasing resistance to chemotherapeutic drugs, alleviating the adverse effects of chemotherapy, minimizing the risk of tumor lysis syndrome, and detoxifying the body from certain chemotherapeutics (Sak 2012). While our study observed a predominant weight loss trend in all patients following diagnosis, this phenomenon was notably more pronounced in men than women. Interestingly, Jung, Kim, and Chung (2020) found no significant changes in weight, body composition, or physical activity during adjuvant chemotherapy among participants with breast cancer. Conversely, Detopoulou et al. (2022) reported reductions in lean mass among chemotherapy recipients without corresponding decreases in body weight. Additionally, the Resting Metabolic Rate decreased without a concurrent reduction in lean mass in patients undergoing chemotherapy and immunotherapy, as highlighted by Detopoulou et al. (2022). Furthermore, Le‐Rademacher et al. (2020) conducted a study involving cancer patients undergoing chemotherapy, revealing that those experiencing a weight loss of 2% or more exhibited significantly poorer overall survival rates than those other participants. Similarly, less than 2% weight loss was linked to worse progression‐free survival in their analysis.

In our study, the mean N‐SAS score, representing patients' chemotherapy‐related symptoms, was 2.16 ± 0.80, with no significant difference observed between women and men. Notably, according to this scale, almost half of the patients reported poor well‐being (42.8%, *n* = 65). Can and Aydiner (2011), in their study with 374 chemotherapy‐receiving cancer patients, reported varying levels of N‐SAS scores: 27.5% reported very good scores, 47.6% reported good scores, 23% reported moderate scores, and 1.9% reported poor scores, with none reporting very poor scores. Stratifying by cancer type revealed that patients with head and neck cancer had the highest average N‐SAS score, suggesting a more negative prognosis in this subgroup. Consistent with findings (Gandhi et al. 2014; Allen‐Ayodabo et al. 2019), patients with head and neck cancer exhibited the highest symptom burden during cancer treatment, emphasizing the need for tailored supportive care interventions in this population. Head and neck cancer encompasses neoplastic growths of the oral cavity, oropharynx, hypopharynx, and lymph nodes in the neck. The specific location of the tumor has a direct impact on the patient's ability to ingest food or liquids, taste perception, appetite, and weight loss (Zebralla et al. 2021). Furthermore, depending on treatment, up to 85% develop severe oral mucositis, which often results in a range of feeding‐related symptoms such as mouth pain and difficulty swallowing (dysphagia) (Chang et al. 2022). A review also has documented a prevalence of malnutrition among this patient population ranging from 25%–65% (Alshadwi et al. 2013). Our findings indicate that the highest weight loss and symptom scores in patients with this type of cancer coincide with these observations and are consistent with the literature.

We observed a significant negative correlation between chemotherapy‐related symptoms and the DPI among all cancer patients, as well as in specific cancer types such as lung, breast, and GIS cancers. This finding suggests that a higher intake of phytochemically rich foods may alleviate chemotherapy‐related symptoms. Supporting this notion, Liu et al. (2021) highlighted the potential of representative phytochemicals to mitigate chemotherapy and radiotherapy side effects through various biological activities, including antioxidation, antimutagenesis, anti‐inflammation, myeloprotection, and immunomodulation. Moreover, several studies have reported inverse associations between higher levels of total flavonoids or specific types of flavonoids and various cancers, including lung (Knekt et al. 1997; Shimazu et al. 2010; Tan et al. 2010), breast (both premenopausal and postmenopausal) (Iwasaki et al. 2008; Lee et al. 2009; Sun and Liu 2006; Verheus et al. 2007; Wei et al. 2020; Wu et al. 2008; Yamamoto et al. 2003), gastric (Sasazuki et al. 2008), prostate (Bolat et al. 2023; Kurahashi et al. 2008; Ozasa et al. 2004), pancreatic (Nöthlings et al. 2007), epithelial ovarian (Cassidy et al. 2014; Gates et al. 2007), and colorectal cancers (Bentyaghoob et al. 2023; Ricciardiello, Bazzoli, and Fogliano 2011; Terry et al. 2002; Yuan et al. 2007). In contrast to the aforementioned studies, our study takes a comprehensive approach to dietary assessment, considering not only the specific bioactive components but also the phytochemical load of the diet. Such an integrated analysis reinforces the crucial role of nutrition in the management of these patients. Also, these findings underscore the potential protective effects of phytochemical‐rich diets against cancer development and treatment‐related symptoms.

Ghoreishy et al. (2021) conducted a case–control study in Iran involving 350 breast cancer patients and 700 controls, utilizing McCarty's method to calculate the DPI consistent with our study; they considered whole grains, fruits, vegetables (excluding potatoes), natural fruit and vegetable juices, tomato sauces, soy products, nuts, legumes, seeds, olives, and olive oil as phytochemically rich foods. Despite similarities, our study revealed a lower average DPI than the study in Iran which may stem from variations in their methodologies including sample size and subject characteristics. Consistent with the findings of Ghoreishy et al. (2021), a higher intake of phytochemical‐rich foods was associated with a reduced likelihood of breast cancer. Another case–control study by Bentyaghoob et al. (2023) in Iran examined the relationship between oxidative balance score and DPI in colorectal cancer patients. Their results highlighted the protective association of a diet rich in phytochemicals and antioxidants against colorectal cancer risk, mainly fruits, vegetables, and whole grains.

Furthermore, in our study, an examination of DPI specifically for cancer revealed that the lowest score was observed in head and neck cancers, while the highest score was observed in gynecological cancers. The lower DPI in head and neck cancer patients may be due to the physical and functional challenges these patients face, such as dysphagia or chewing, which are common side effects of both the disease and its treatments (e.g., chemoradiation therapy) (Zebralla et al. 2021). These issues could lead to a reduced intake of fruits, vegetables, and whole grains‐foods that are rich in phytochemicals‐resulting in a lower overall DPI. Additionally, these patients may rely more on softer, processed, or nutrient‐dense foods that are easier to consume but lower in phytochemical content. On the other hand, the higher DPI in gynecological cancer patients may be related to fewer dietary restrictions and greater flexibility in food choices during treatment (Obermair et al. 2017). Many gynecological cancer patients may retain the ability to consume a broader range of plant‐based foods, which could contribute to a higher intake of fruits, vegetables, legumes, and whole grains, thus resulting in a higher DPI.

Despite the valuable insights provided by this study, several limitations should be acknowledged. Firstly, the study sample may not represent all cancer patients, as it was conducted at a single institution and included a relatively small number of participants particularly for some specific cancers (*n* < 10), such as head and neck and gynecological cancers. This limits the statistical power of the tests that can be conducted. Additionally, the retrospective nature of the study design may introduce biases related to data collection and analysis. Furthermore, the use of self‐reported measures, such as the N‐SAS and DPI, may be subject to recall bias and social desirability bias, potentially affecting the accuracy of the results. Another limitation is the lack of data on foods containing hormones or xenoestrogens, which may influence cancer outcomes, especially in breast cancer patients. Furthermore, the absence of detailed information on the types of food groups consumed by the participants limits our ability to fully interpret the DPI.

Finally, DPI examines the possible health effects of foods rich in phytochemicals. More comprehensive methodologies are required to examine cancer‐specific phytochemicals. Future research incorporating larger, more diverse samples and utilizing objective measures of symptom burden and dietary intake is needed to validate and expand upon the findings of this study.

## 5 Conclusion

In conclusion, our study adds to the existing evidence supporting the role of dietary phytochemicals in symptom management and treatment outcomes among cancer patients undergoing chemotherapy. While further research is needed to fully elucidate the mechanisms underlying these associations, our findings underscore the importance of dietary interventions as adjunctive therapies in cancer care. Healthcare providers should consider gender and cancer type when developing personalized care plans for cancer patients, with particular attention to symptom management and nutritional support. Moreover, interdisciplinary collaboration between oncologists, dietitians, and researchers is essential for developing tailored dietary interventions to optimize patient outcomes in oncology settings.

## Author Contributions

**Barış Yüksel:** conceptualization (equal), data curation (equal), investigation (equal), visualization (equal), writing – original draft (equal). **Gözde Dumlu Bilgin:** conceptualization (lead), data curation (lead), investigation (equal), visualization (equal), writing – original draft (equal). **Hasan Kaan Kavsara:** software (equal), supervision (equal), visualization (equal), writing – original draft (equal), writing – review and editing (equal).

## Conflicts of Interest

The authors declare no conflicts of interest.

## Data Availability

Data will be made available on request.
